# Electrolyte imbalance and liver function test abnormalities among pregnant women with hyperemesis gravidarum at Wag-himra zone public hospitals, Northeast Ethiopia, 2023: a comparative cross-sectional study

**DOI:** 10.3389/fmed.2024.1451036

**Published:** 2024-12-17

**Authors:** Abebaw Worede, Teshiwal Deress, Habtamu Wondifraw, Getnet Fetene, Alemseged Berie

**Affiliations:** ^1^Department of Clinical Chemistry, School of Biomedical and Laboratory Sciences, College of Medicine and Health Sciences, University of Gondar, Gondar, Ethiopia; ^2^Department of Quality Assurance and Laboratory Management, School of Biomedical and Laboratory Sciences, College of Medicine and Health Sciences, University of Gondar, Gondar, Ethiopia

**Keywords:** hyperemesis gravidarum, electrolyte imbalances, liver function test abnormalities, pregnant women, Wag-himra zone, public hospitals

## Abstract

**Background:**

Hyperemesis gravidarum affects about 4.8% of pregnant women. It can lead to electrolyte imbalances and liver function disturbances, which may result in pregnancy complications. Despite its prevalence, data on these abnormalities in the study area is scarce. Therefore, the current study investigated these health concerns among HG-affected pregnant women in this region from June to October 2023.

**Method:**

A comparative cross-sectional study was conducted on 123 study participants. Socio-demographic, clinical, and anthropometric data were collected using structured questionnaires. Blood samples were taken to determine liver function tests and electrolyte levels. Statistical analysis was performed using a one-way ANOVA with a Tuckey *post hoc* test, Kruskal-Wallis with a Mann–Whitney *U* test, and binary logistic regression analysis. A *p*-value of <0.05 with 95% confidence intervals was considered statistically significant.

**Results:**

The results showed that pregnant women with hyperemesis gravidarum had significantly higher levels of AST and ALT and lower levels of Na+ and K+ compared to normal pregnant women and non-pregnant women. The abnormalities observed were elevated AST (48.8%), ALT (46.3%), and decreased Na+ (51.2%) and K+ (41.5%). Hyperemesis gravidarum was associated with both electrolyte imbalances and liver function test abnormalities.

**Conclusion:**

Pregnant women with hyperemesis gravidarum experience electrolyte imbalances and liver function test abnormalities. Given the marked increase in liver enzymes and decrease in electrolyte levels, the authors recommend routine laboratory testing of liver function and electrolyte parameters for pregnant women with hyperemesis gravidarum is recommended.

## Introduction

Hyperemesis gravidarum (HG) is severe nausea and repeated vomiting that prevents oral intake of food and leads to dehydration and ketoacidosis during pregnancy (1). During the first trimester, around 70–80% of pregnant women experience nausea and vomiting. The symptoms typically start around the 4th to 6th week of gestation, peak during weeks 8 to 12th weeks, and settle by the 20th week. The disease ranges from mild to severe illness and is termed nausea and vomiting of pregnancy (NVP) (2–4). The most severe and persistent form of NVP is known as HG, it alludes to the state of severe, intractable nausea and emesis that requires medical intervention (5, 6). Hyperemesis gravidarum affects about 0.5 to 4.8% of pregnant women and is the second most common indication for admission in pregnancy for women with live births (7, 8).

The complications of HG are dehydration, remarkable ketonuria, a loss of body weight of >5%, electrolyte imbalance, and abnormal liver function tests (LFT) (9–11). If left untreated, HG can lead to Wernicke’s encephalopathy (12–14), central pontine myelinolysis, and liver dysfunction. Serum electrolyte imbalances in patients with hyperemesis may result in severe hypokalemia. Potassium abnormalities have been reported to increase the mortality in pregnant women with hyperemesis (15). Besides, severe hypokalemia may cause rhabdomyolysis in the setting of HG (7). Common findings of HG include elevated liver enzymes, electrolyte imbalances, and/or ketonuria on urinalysis (16). Electrolyte imbalances due to severe HG induce cardiac and neuromuscular impairment; at the same instant, liver abnormalities and nutritional deficiency have even recently led to maternal death (15, 17, 18).

Hyperemesis gravidarum is the leading cause of hospitalization in the first trimester and the second most common indication for pregnancy hospitalization next to pre-matured labor during pregnancy. It causes potentially life-threatening complications and is potentially lethal if not treated (19–21). Globally, approximately 0.5 to 4.8% of pregnant women developed HG during their pregnancy (22). Even though this devastating health problem is huge with a significant adverse outcome, there is limited data regarding the electrolyte imbalances and liver function test abnormalities among pregnant women with HG in Ethiopia. While numerous studies have explored this topic with varying results across different countries, many Ethiopian women experiencing emesis in the early weeks of gestation are often left untreated, as these symptoms are generally seen as a normal part of pregnancy. Our study aims to investigate the prevalence of biochemical abnormalities in this population and to compare the mean levels of these biochemical markers. This may be used to reduce maternal and fetal morbidity and mortality due to complications of electrolyte imbalances and liver function test abnormalities in HG and improve the quality of life by enhancing health education and related information about the nature of disease and control mechanisms.

## Methods

### Study area, design, and period

A comparative cross-sectional study was conducted in Wag-himra zone, located in northeast Ethiopia, from June to October 23, 2023. Wag-himra zone is one of the 14 zones in the Amhara National Regional State, with an approximate population of 426,038 as of the 2007 census. Within this zone, there are three public hospitals: Tefera Hailu General Memorial Hospital, Amdework Primary Hospital, and Ziquala Primary Hospital, along with 34 health centers. The capital city of Wag-himra zone is Sekota, situated 720 km from Addis Ababa (the capital city of Ethiopia) and 435 km from Bihar Dar (the capital city of the Amhara region).

### Populations

In the study, the population consisted of two groups: cases and controls. The cases included pregnant women who were admitted and diagnosed with hyperemesis gravidarum in the study hospitals during the study period and who fulfilled the eligibility criteria. On the other hand, the controls comprised pregnant women without hyperemesis gravidarum who visited the ANC, as well as non-pregnant women who visited the study hospitals during the study period for caregiving for their families.

### Inclusion and exclusion criteria

The study recruited pregnant women without gestational hyperglycemia and age- and trimester-matched non-pregnant women visiting the study hospitals during the study period. All participants were willing to participate, provided information, had no history of diabetes, HIV/AIDS, hepatitis, or liver/kidney problems, and showed no current symptoms. For non-pregnant women, a negative pregnancy test was required. The study excluded participants with mental health issues, hearing impairments, or any serious health condition limiting their ability to provide accurate information. Additionally, those with pre-existing conditions affecting electrolytes or liver function (diabetes, HIV/AIDS, hepatitis, or previous liver/kidney problems) were excluded. The same applied to individuals taking medications known to influence these tests (e.g., acetaminophen, allopurinol, anti-TB medications, statins, antibiotics, antivirals, or thiazide diuretics). Exclusion was confirmed using a checklist, medical record review, and current HIV/AIDS and hepatitis status verification.

### Operational definitions

#### Hyperemesis gravidarum

A severe and persistent nausea and excessive vomiting during pregnancy that starts before the end of the 22nd week of gestation, subdivided into mild and severe categories (5).

#### Liver function test abnormalities

Considered abnormal when at least one of AST, ALT, albumin, TBIL, and ALP falls above the upper limit of normal in the reference range (23, 24).

#### Electrolyte imbalances

An altered electrolyte balance is indicated by a high or low level of electrolytes in at least one of the test results. Specific concentration ranges define hyponatremia, hypernatremia, hypokalemia, hyperkalemia, hypochloremia, and hyperchloremia for sodium, potassium, and chloride, respectively (25, 26).

#### Body mass index (BMI)

BMI is categorized as underweight (<18.5 kg/m^2^), normal (18.5–24.9 kg/m^2^), overweight (25–29.9 kg/m^2^), and obese (≥30 kg/m^2^) (27).

#### Pregnancy trimesters

A period of 3 months; the first trimester is up to 13 weeks, the second trimester is from 14 to 27 weeks or 4–6 months, and the third trimester is beyond 27 weeks or 7–9 months of pregnancy (1, 28).

#### Parity

The number of deliveries with a gestational age of 28 weeks or more, regardless of whether the child was born alive or was stillborn (29).

#### Gravidity

The total number of times a woman has been pregnant, including all live births, pregnancies terminated at less than 6 months, or miscarriages (30).

### Sample size and sampling technique

The sample size was calculated using mean difference values in openEpi software version 2.3, assuming a 95% confidence interval, 80% power, mean values of potassium(gives the maximum sample size) with the HG cases (3.95 ± 0.24) and controls (3.79 ± 0.35) mEq/L and a proportion of HG cases and controls of 1:2. The sample size in each group was calculated using the formula: [(s12 + s22) f (*α*, *β*)]/(μ1-μ2)2 for each assessment of liver function biomarkers and electrolytes, and then the maximum sample size was 123 with 10% non-response rate The convenience sampling technique was used to recruit study cases of HG and controls until the required maximum sample size of a total of 123 study participants was fulfilled. All cases that were diagnosed and documented on the chart were selected with daily monitoring of all new admissions until the sample size was fulfilled in the study hospitals. For each case, one pregnant woman without HG age and trimester-matched and non-pregnant age-matched control groups were recruited on the same day of data collection. The sample size was also allocated for each of the three study hospitals based on 3 months of HG reports (January to March 2023): 22 from Tefera Hailu Memorial General Hospital, 16 from Amdework, and 14 from Ziquala Hospitals. Therefore, the sample size allocated for each hospital was 17, 13, and 11, Tefera Hailu Memorial General Hospital, Amdework Hospital, and Ziquala Hospital, respectively, based on the population allocation formula of nj = n*Nj/N. Where nj = is the sample size of the jth stratum; *n* = n1 + n2 + … + nk is the total sample size; Nj = is the population size of the jth stratum; and *N* = n1 + n2 + … + nk is the total population size.

### Data collection

Socio-demographic and clinical characteristics data were collected from study participants using structured questionnaires face-to-face and checklists after pre-testing in 5% of the participants. The questionnaire was adapted from previous literature (31–33). Originally, the questionnaire was prepared in English and translated into local languages (Amharic and Agewigna) by two independent researchers to observe its consistency. Data were collected by trained midwives. For electrolyte and liver function tests, venous blood samples of 5 mL were collected using a sterile syringe and then transferred to a serum separation tube by laboratory technologists. Then, the samples were allowed to clot for 30 min and centrifuged at 3000 rpm for 5 min to obtain the serum. The serum was separated into sterile tubes. Electrolyte imbalance and liver function test abnormalities were measured by an American-made Siemens EXL 200 chemistry analyzer.

### Blood sample collection and processing

Data collectors received training on data collection procedures, including obtaining informed consent, patient approach, questionnaire administration, and research ethics. Daily meetings were held between investigators and data collectors to discuss challenges, assess performance, and monitor progress on data collection. Following written informed consent and questionnaire completion, participants received a unique serial number. Laboratory professionals then collected a 5 mL venous blood sample. The collected blood samples were coded and stored at room temperature for 30 min to allow coagulation. Subsequently, the samples were transported to the clinical chemistry section and centrifuged at 3,000 rpm for 5 min to separate serum for analysis. After centrifugation, the serum was transferred to a nuc tube and stored in a refrigerator at 2–8°C until analysis. All activities, including patient identification, preparation, blood sample collection, transportation, and storage, adhered to good laboratory practices. Quality controls were performed and verified before running participant samples to confirm the analytical performance and functionality of the instrument. Additionally, investigators closely monitored and frequently checked the laboratory analysis to ensure completeness and consistency. All results were carefully recorded in designated spaces, referencing the labeling attached to the questionnaires.

### Data analysis and interpretation

Data were entered into EpiData 4.6 and exported to SPSS version 27 for analysis. Frequencies and percentages of participants were calculated. Bivariable and multivariable logistic regression models assessed the association between dependent and independent variables. Variables with *p* ≤ 0.25 in the bivariable analysis were included in multivariable logistic regression analysis. Pregnant women with and without HG and non-pregnant women were compared using one-way ANOVA, followed by Tukey’s *post hoc* test, and the Kruskal-Wallis test, followed by Mann–Whitney U test for normally and non-normally distributed data, respectively, to identify mean differences in biochemical and anthropometric parameters. The normality of variables was assessed using Kolmogorov–Smirnov and Shapiro–Wilk tests, Skewness (−1, +1), and Kurtosis (−3, +3), and presented as mean ± SD for parametric tests and median (interquartile range) for non-parametric tests. Statistical significance was set at a 95% confidence interval with a *p*-value <0.05. Results were presented using tables, graphs, and narrative text.

### Ethical clearance

Ethical clearance with approval reference number (SBMLS/523) was obtained from the Research and Ethical Review Committee of the School of Biomedical and Laboratory Sciences, College of Medicine and Health Sciences, University of Gondar. Then permission letters were obtained from Wag-himra Zone public hospitals. Data were collected after written informed consent was obtained from each study participant. Confidentiality was maintained using codes so that the names and any identifiers of participants were not used on the questionnaire and laboratory requests. Participants with abnormal test results were communicated through data collectors and linked to hospitals for treatment purposes.

## Results

### Socio-demographic, obstetrics, and clinical characteristics of study participants

This study included 123 participants, with mean ages of 26.56 ± 4.96, 26.24 ± 4.58, and 26.73 ± 4.75 years for pregnant women with HG (*n* = 41), pregnant women without HG (*n* = 41), and non-pregnant women (*n* = 41), respectively. The majority, 108 (87.8%), were in the 20–35 age group. Among the participants, 42 (34.1%) had no formal education, 36 (29.3%) of the pregnant women were in their first trimester, 73 (59.3%) lived in urban areas, and only 6 (14.6%) experienced severe vomiting episodes (≥13 times) (Table 1).

**Table 1:** Socio-demographic and obstetrics characteristics of study participants at Wag-himra District public hospitals, northeast Ethiopia, 2023 (*n* = 123).

	Categories	HG cases (41)	Normal-pregnant (41)	Non-pregnant (41)	Total (123)	*p*-value (chi-square)
		*n* (%)	*n* (%)	*n* (%)	*n* (%)	
Age	<20	4 (9.8%)	3 (7.3%)	3 (7.3%)	10 (8.1%)	0.967
	20–35	35 (85.4)	37 (90.2%)	36 (87.8%)	108 (87.8%)	
	>35	2 (4.9%)	1 (2.4%)	2 (4.9%)	5 (4.1%)	
Marital status	Single	4 (9.8%)	7 (17.1%)	11 (26.8%)	22 (17.9%)	0.409
	Married	26 (63.3%)	21 (51.2%)	18 (43.9%)	65 (52.8%)	
	Divorced	4 (9.8%)	6 (14.6%)	5 (12.2%)	15 (12.2%)	
	Widowed	7 (17.1%)	7 (17.1%)	7 (17.1%)	21 (17.1%)	
Educational status	No-formal education	12 (29.3%)	17 (41.5%)	13 (31.7%)	42 (34.1%)	0.275
	Primary education	5 (12.2%)	2 (4.9%)	3 (7.3%)	10 (8.2%)	
	Secondary education	9 (22.0%)	12 (29.3%)	8 (19.5%)	29 (23.6%)	
	College and above	15 (36.6%)	10 (24.4%)	17 (41.5%)	42 (34.1%)	
Residence	Urban	24 (58.5%)	22 (53.7%)	27 (65.9%)	73 (59.3%)	0.56
	Rural	17 (41.5%)	19 (46.3%)	14 (34.1%)	50 (40.7%)	
Occupations	Housewife	13 (31.7%)	8 (19.5%)	7 (17.1%)	28 (22.8%)	NA
	Student	3 (7.3%)	6 (14.6%)	5 (12.2%)	14 (11.4%)	
	Marchant	9 (22.0%)	13 (31.7%)	7 (17.1%)	29 (23.6%)	
	Government employee	8 (19.5%)	5 (12.2%)	14 (34.1%)	27 (22.0%)	
	Others	8 (19.5%)	9 (22.0%)	8 (19.5%)	25 (20.3%)	
Gravidity	Primigravida	13 (31.7%)	8 (19.5%)	10 (24.4%)	31 (25.2%)	0.17
	Multigravida	28 (68.3%)	33 (80.5%)	31 (75.6%)	92 (74.8%)	
Parity	Nulliparous	7 (17.1%)	8 (19.5)	15 (36.6%)	30 (24.4%)	0.955
	Primiparous	4 (9.8%)	15 (36.6)	6 (14.6%)	25 (20.3%)	
	Multiparous	30 (73.1%)	18 (43.9%)	20 (48.8%)	68 (55.3%)	
Gestational age in weeks (Trimesters)	1st trimester (≤13 weeks)	16 (39.1%)	20 (48.8%)	-	36 (43.9%)	0.959
	2nd trimester (14-27 weeks)	11 (26.8%)	10 (24.4%)	-	21 (25.6%)	
	3rd trimester (>27 weeks)	14 (34.1%)	11 (26.8%)	-	25 (30.5%)	
SBP	≤90	19 (46.3%)	3 (7.3%)	0 (0%)	22 (17.9%)	0.001
	>90	22 (53.7%)	38 (92.7%)	41 (100%)	101 (82.1%)	
DBP	≤60	20 (48.8%)	5 (12.2%)	0 (0%)	25 (20.3%)	0.001
	>60	21 (51.2%)	36 (87.8%)	41 (100%)	98 (79.7%)	
Vomiting episodes	Mild (<7)	13 (31.7%)	-	-	13 (31.7%)	-
	Moderate (7–12)	22 (53.7%)	-	-	22 (53.7%)	
	Sever (≥13)	6 (14.6%)	-	-	6 (14.6%)	

HG, hyperemesis gravidarum; SBP, systolic blood pressure; DBP, diastolic blood pressure, and NA, are not applicable.

### Comparison of electrolyte and liver function test parameters across study groups

Statistical analysis was performed using a 1-way ANOVA followed by a Tuckey *post hoc* test and a Kruskal-Wallis H test, followed by a Mann–Whitney *U* test for normally and non-normally distributed data for comparison groups, respectively. In a Tukey *post hoc* pairwise comparison test the mean level of AST was found to be significantly higher in pregnant women with HG compared to both normal pregnant women and non-pregnant women (*p* < 0.001). Conversely, the mean level of Na+ was significantly decreased in pregnant women with HG compared to normal pregnant women and non-pregnant women (*p* < 0.001) (Table 2).

**Table 2:** A one-way ANOVA analysis to compare the electrolytes and liver function test parameters of study participants at Wag-Himra Zone public hospitals, northeast Ethiopia, 2023 (*n* = 123).

Parameters	Non-pregnant women (41)	Normal pregnant women (41)	Women with HG (41)	*p* value	^X^ *p* value	^Y^*p* value	^Z^*p* value
	Mean ± SD	Mean ± SD	Mean ± SD				
AST (IU/L) *^α^*	46.22 ± 6.11	59.02 ± 8.77	70.27 ± 8.75	<0.001*	<0.001*	<0.001*	<0.001*
ALT (IU/L) ^α^	43.29 ± 9.66	43.46 ± 9.41	67.29 ± 7.72	<0.001*	<0.001*	0.996	<0.001*
DBil (mg/dl) ^α^	0.34 ± 0.20	0.28 ± 0.16	0.38 ± 0.17	0.035*	0.553	0.263	0.027*
TBil (mg/dl) *^α^*	0.77 ± 0.53	0.79 ± 0.57	0.88 ± 0.47	0.554	0.567	0.983	0.676
TP (g/dl) ^α^	6.98 ± 1.10	7.27 ± 1.18	7.68 ± 1.28	0.043*	0.036*	0.633	0.252
Na^+^ (mg/dl) ^α^	137.45 ± 1.34	119.97 ± 5.51	80.21 ± 14.04	<0.001*	<0.001*	<0.001*	<0.001*
K^+^ (mg/dl) ^α^	3.97 ± 0.41	3.95 ± 0.73	2.82 ± 0.47	<0.001*	<0.001*	0.968	<0.001*
Cl^−^ (mg/dl) ^β^	100.40 (10.97)	100.59 (11.53)	100.06 (29.70)	0.089	0.058	0.295	0.096
ALP (IU/L) ^β^	60.0 (50)	80.0 (49)	60.0 (36)	0.130	0.806	0.072	0.095
Alb (g/dl) ^β^	4.13 (1.14)	3.84 (1.84)	3.87 (1.21)	0.788	0.489	0.714	0.752

AST, Aspartate Aminotransferase; ALT, Alanine Transaminase; ALP, Alkaline Phosphatase; DBil, Direct Bilirubin; TBil, Total Bilirubin; TP, Total Protein; Na^+^, Sodium; K^+^, Potassium; Cl^−^, Chloride; Alb, albumin; SBP, Systolic Blood Pressure; and DBP, Diastolic Blood Pressure.

α Data expressed as mean ± SD.

β Data expressed as median (IQR).

*p* value-comparison among HG cases, normal pregnant and non-pregnant, ^X^*p* value-comparison of HG cases and non-pregnant in a pairwise comparison test; ^Y^*p* value-comparison of normal pregnant to non-pregnant in a pairwise comparison test; ^Z^*p* value-comparison of HG cases to normal pregnant in a pairwise comparison test; and * = statistically significant mean or median difference due to *p* < 0.05.

### Magnitude of electrolyte imbalance and liver function test abnormalities

**Figure 1:**
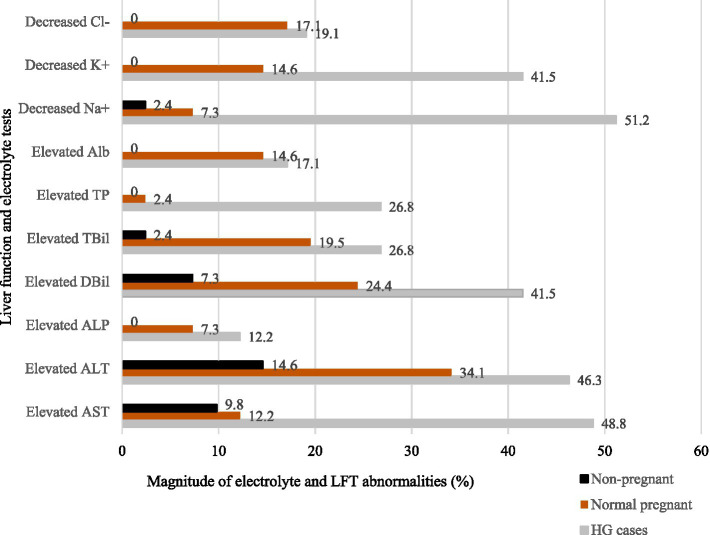
Bar graph of the abnormalities in electrolytes and liver function test parameters among study groups at Wag-Himra Zone public hospitals, northeast Ethiopia, 2023 (*N* = 123).

The magnitude of electrolyte imbalances and abnormal LFTs varied among the study groups. According to the graph, the highest level of AST was 48.8%, and there was a notable decrease in Na+ levels, with the lowest recorded at 51.2% (Figure 1).

### Factors associated with liver function test abnormalities among pregnant women participants

In the multivariable analysis, the status of HG, gestational age, and parity were factors found to be the dominant factors of liver function abnormalities among pregnant women study participants. The odds of having LFT abnormalities were 6.1 times higher among participants with HG compared to normal pregnant women (AOR = 6.1; 95% CI: 1.49, 24.72; *p* = 0.012). The odds of having LFT abnormalities were 13.6 times higher in the first trimester (AOR = 13.6; 95% CI: 2.96, 62.31; *p* < 0.001) and 7.0 times higher in the second trimester (AOR = 7.0; 95% CI: 1.30, 38.03; *p* = 0.024) compared to those in the third trimester (Table 3).

**Table 3:** Bivariable and multivariable logistic regression analysis of maternal clinical factors associated with liver function test abnormalities among pregnant women at Wag-Himra Zone public hospitals, northeast Ethiopia, 2023 (*n* = 82).

Variables	Categories	LFT abnormalities		COR 95% CI	AOR 95% CI	*p*-value
		Yes *n* (%)	No *n* (%)			
Status of HG	HG cases	23 (56.1%)	18 (43.9%)	2.2 (0.83, 4.81)	6.1 (1.49, 24.72)	0.012*
	Normal pregnant	16 (39.0%)	25 (61.0%)	1	1	-
Gestational age (trimesters)	1st trimester	24 (66.7%)	12 (33.3%)	8.0 (2.41, 26.57)	13.6 (2.96, 62.31)	<0.001*
	2nd trimester	10 (47.6%)	11 (52.4%)	3.6 (0.99, 13.36)	7.0 (1.30, 38.03)	0.024*
	3rd trimester	5 (20.0%)	20 (80.0%)	1	1	-
BMI	Underweight (<18.5)	10 (31.3%)	22 (68.8%)	0.3 (0.13, 0.8)	0.4 (0.09, 1.36)	0.132
	Normal weight (18.5–24.99)	29 (58.0%)	21 (42.0%)	1	1	-
Gravidity	Primigravida	13 (61.9%)	8 (38.1%)	2.2 (0.79, 6.05)	5.3 (0.90, 30.89)	0.065
	Multigravida	26 (42.6%)	35 (57.4%)	1	1	-
Parity	Nulliparious	3 (20.0%)	12 (80%)	0.3 (0.07, 1.18)	0.3 (0.04, 2.03)	0.204
	Primiparous	14 (73.7%)	5 (26.3%)	3.3 (1.03, 10.64)	8.2 (1.54, 43.40)	0.013*
	Multiparous	22 (45.8%)	26 (54.2%)	1	1	-

LFT, Liver Function Test; BMI, Body Mass Index; HG, Hyperemesis Gravidarum; AOR, Adjusted Odds Ratio; COR, Crude Odds Ratio, CI, Confidence Interval; * = significant at *p* < 0.05.

### Factors associated with electrolyte imbalance among pregnant women participants

In the multivariable logistic analysis, the status of HG was significantly associated with electrolyte imbalances. The odds of experiencing electrolyte imbalances were 9.0 times higher (AOR = 9.0; 95% CI: 2.60–30.75) (*p* < 0.001) among pregnant women with HG compared to those without HG (Table 4).

**Table 4:** Bivariable and multivariable logistic regression analysis of maternal clinical factors associated with electrolyte imbalances among pregnant women at Wag-Himra Zone public hospitals, northeast Ethiopia, 2023 (*n* = 82).

Variables	Categories	Electrolyte imbalance		COR 95% CI	AOR 95% CI	*p*-value
		Yes *n* (%)	No *n* (%)			
Status of HG	HG cases	25 (61.0%)	16 (39.0%)	9.2 (3.00, 28.21)	9.0 (2.60, 30.75)	<0.001*
	Normal pregnant	5 (12.2%)	36 (87.8%)	1	1	-
BMI	Underweight (<18.5)	14 (43.8%)	18 (56.3%)	2.0 (0.79, 5.08)	1.2 (0.39, 3.36)	0.805
	Normal (18.5–24.99)	14 (28.0%)	36 (72.0%)	1	1	-
SBP	≤90	11 (50.0%)	11 (50.0%)	2.5 (0.92, 6.92)	0.9 (0.11, 7.24)	0.929
	>90	17 (28.3%)	43 (71.7%)	1	1	-
DBP	≤60	12 (46.2%)	14 (53.8%)	2.1 (0.82, 5.62)	1.2 (0.15, 7.75)	0.938
	>60	16 (28.6%)	40 (71.4%)	1	1	-

AOR, Adjusted Odd Ratio; COR; Crude Odd Ratio; CI, Confidence Interval; BMI, Body Mass Index; HG, Hyperemesis Gravidarum; * = significant at *p* < 0.05.

## Discussion

Hyperemesis gravidarum can lead to significant complications, including electrolyte imbalances and abnormal liver function tests. Despite its prevalence, limited research exists on the extent of these complications in resource-limited settings like the Wag-himra Zone in northeast Ethiopia.

Our study found statistically significant increases in mean AST levels in HG cases compared to non-pregnant women and pregnant women without HG, consistent with previous findings from a Malaysian study. In contrast, studies in Iraq, Israel, and India did not show significant differences in AST levels between normal and HG pregnant women. Additionally, our findings revealed significantly higher ALT levels in HG cases compared to both non-pregnant women and normal pregnant women, which aligns with results from an Israeli study. In contrast, research from Iraq, Turkey, and India reported varying ALT levels (34–38). These discrepancies may be attributed to differences in nutrition, lifestyle, study design, and exclusion criteria. For example, the Iraqi study excluded women with trophoblastic disease, while the Israeli and Indian studies excluded women with a BMI below 18.

A significant difference in the mean level of direct bilirubin was found when comparing normal pregnant women with HG cases. The reason for this elevation in direct bilirubin specifically in HG cases remains unclear, but it could be due to malnutrition, intrahepatic cholestasis, or a decrease in hepatic bilirubin clearance (bile clearance defect) leading to a buildup of direct bilirubin in the body (39, 40). Additionally, this study found a statistically significant elevation in total protein in hyperemesis cases compared to the non-pregnant women. However, the comparison of hyperemesis cases against normal pregnant women reported elsewhere was not significantly different (35).

The current study found significant decreases in sodium (Na+) levels in non-pregnant women compared to both normal pregnant women and HG cases. Normal pregnant women also had lower Na+ levels compared to HG cases. These findings align with studies from Iraq and Saudi Arabia (35, 41), but contradict reports from Turkey and Israel (36, 42). Similarly, potassium (K+) levels were significantly lower in HG cases compared to non-pregnant women and normal pregnant women. However, there was no significant difference between non-pregnant and normal pregnant women. Again, these results were consistent with findings from Iraq and Saudi Arabia (35, 41), while studies in Turkey (42) and Israel (36) did not report a decrease, which may be attributed to differing study methodologies and exclusion criteria.

In the current study, elevated AST was found at 48.8%, higher than those reported in India and France (37, 43). However, it was lower than the findings reported in the Netherlands (44). Similarly, the magnitude of elevated ALT was 46.3%, exceeding values reported in India and France (37, 43). This difference is possibly explained by geographical variation, nutritional status (study participants in the present study might be malnourished due to our country’s lower economic development compared to France and India), and study design differences (the studies in France and India were retrospective).

Hypoalbuminemia (17.1%) from the current findings was lower than those reported in Brazil (45). This difference might be related to lifestyle factors. The possible reason for the lower albumin result is due to a combined effect of hypovolemia and malnutrition in HG, potentially linked to vomiting severity (46, 47). This study found hyponatremia (51.2%) and hypokalemia (41.5%), consistent with results in Brazil (45). However, it reported higher rates of hypokalemia and lower rates of hyponatremia compared to a French study on normal pregnant and hyperemetic women (43). This dissimilarity is likely attributed to variations in ethnicity and lifestyle factors (living conditions in France might be more developed with more advanced healthcare compared to Ethiopia), along with differences in study design (the French study is a retrospective cohort study).

In the current study, HG was significantly associated with abnormalities in liver function tests. The odds of having abnormalities in liver function tests were 6.08 times higher in the HG cases compared to normal pregnant women. The exact relationship between liver function test abnormalities and HG is not well understood. However, it is believed to be related to the increased stress on the liver caused by excessive vomiting and dehydration (48).

This study also revealed that gestational age in weeks during the first and second trimesters was statistically significantly associated with liver function test abnormalities. The odds of having liver function test abnormalities were 13.6 and 7.0 times higher in the first and second trimesters, respectively, compared to the third trimester. This could be explained by the body’s reaction to the pregnancy hormone, particularly human chorionic gonadotropin, which is produced in higher amounts during the first and second trimesters than in the third trimester. Additionally, the placenta is known to be a source of TNF-*α*. The rise in this inflammatory cytokine can be hypothesized to be involved in the pathogenesis of HG, along with liver derangement in pregnant women. Another possible mechanism is that the production of reactive oxygen species, as a result of impaired fatty acid metabolism, can lead to the production of inflammatory cytokines, ultimately leading to HG and liver dysfunction (31, 32, 49).

Primiparous parity was significantly associated with liver function test abnormalities. The odds of having a liver function test abnormality were 8.2 times higher in primiparous women compared to multiparous women. This might be due to exposure to stress for the first time, followed by exposure to severe HG. The degree of derangement in the liver function tests may indicate the severity of vomiting and the elevation of the human chorionic gonadotropin hormone (32).

The status of the HG case was significantly associated with electrolyte imbalances. The odds of having an electrolyte imbalance were 9.0 times higher in HG cases compared to normal pregnant women. This could be due to subsequent nutritional deficiencies causing electrolyte wasting, extracellular fluid volume diminishment, and activation of the renin-angiotensin-aldosterone system. This might be combined with the physiological changes that advance potassium wasting in pregnancy, including volume expansion, increased renal blood flow, increased glomerular filtration rate, and an increase in cortisol, which may contribute to the patient having a significantly low total body potassium level (15, 18, 50, 51).

## Conclusion

Pregnant women diagnosed with HG were found to have significantly higher rates of electrolyte imbalances and abnormal liver function tests compared to control groups. The severity of HG, gestational age at presentation, and parity were identified as factors influencing abnormal liver function tests. Particularly, only HG itself was identified as a determinant for electrolyte imbalances. Pregnant women with HG displayed statistically significant increases in liver enzyme levels and decreases in electrolytes compared to control groups.

## Data Availability

The raw data supporting the conclusions of this article will be made available by the authors, without undue reservation.
